# Systemic Lupus Erythematous and Obstructive Sleep Apnea: A Possible Association

**DOI:** 10.3390/life13030697

**Published:** 2023-03-05

**Authors:** Roni Meidan, Ofir Elalouf, Riva Tauman, Victoria Furer, Ari Polachek, Jonathan Wollman, Tali Eviatar, Michael Zisapel, David Levartovsky, Estelle Seyman, Ori Elkayam, Daphna Paran

**Affiliations:** 1 Department of Rheumatology, Tel Aviv Sourasky Medical Center, Tel Aviv 6423906, Israel; 2 Sackler Faculty of Medicine, Tel Aviv University, Tel Aviv 6997801, Israel; 3 Sleep Medicine Center, Tel Aviv Sourasky Medical Center, Tel Aviv 6423906, Israel; 4 Department of Neurology, Tel Aviv Sourasky Medical Center, Tel Aviv 6423906, Israel

**Keywords:** systemic lupus erythematosus, obstructive sleep apnea, sleep-disordered breathing, WatchPAT

## Abstract

Marked fatigue is common in patients with systemic lupus erythematosus (SLE). This study aimed to assess the association of sleep disorders, including obstructive sleep apnea (OSA), with SLE. Forty-two consecutive patients with SLE and 20 healthy controls were recruited and underwent a one-night ambulatory sleep examination. They completed questionnaires, including the Pittsburgh Sleep Quality Index (PSQI) and Functional Assessment of Chronic Illness Therapy (FACIT). SLE disease activity and damage were assessed by the SLE Disease Activity Index 2000 (SLEDAI-2K) and the Systemic Lupus International Collaborating Clinics/American College of Rheumatology (SLICC/ACR) damage index (SDI). A significantly increased apnea/hypopnea index was noted in the SLE group compared to healthy controls (*p* = 0.004). SLE patients had higher rates of moderate-to-severe OSA (*p* = 0.04), PSQI (*p* = 0.001), and FACIT scores (*p* = 0.0008). Multivariate analysis revealed that the SDI was associated with OSA (*p* = 0.03). There was a positive association between SLEDAI-2K and moderate-to-severe OSA (*p* = 0.03). Patients with SLE had an increased prevalence of OSA and poorer quality of sleep compared to healthy controls. Our findings suggest that active disease and accumulated damage may be associated with OSA. These findings highlight the importance of identifying the presence of OSA in patients with SLE.

## 1. Introduction

Systemic lupus erythematosus (SLE) is a chronic, autoimmune disease that can affect various organ systems in the body and is thought to have both genetic and environmental factors. SLE primarily affects women of childbearing age. Its symptoms are not specific and can vary widely, possibly including arthralgias and arthritis, skin rashes, pleuritis and pericarditis, and renal and neurologic involvement [1].

Fatigue is a common and debilitating symptom experienced by patients with SLE. It is reported to be present in up to 90% of patients and able to have a significant impact on the quality of life [2]. Fatigue in SLE patients can be caused by a variety of factors. It has frequently been associated with depression and other comorbid conditions such as fibromyalgia and also might be closely associated with disease activity and with the presence of sleep disturbances [3,4,5].

Some studies suggested that sleep disturbances correlate with SLE disease activity and the presence of additional symptoms such as pain and steroid use [6]. Importantly, sleep disturbance symptomatology, such as fatigue and increased pain, overlap with constitutional inflammatory symptoms of SLE and may mimic disease-related relapse [7,8].

Individuals with SLE, as well as other rheumatological diseases, including rheumatoid arthritis, ankylosing spondylitis, and psoriatic arthritis, are known to experience sleep disturbances, which can have a significant impact on their overall health and quality of life [9,10,11,12]. These disturbances include reduced sleep efficiency, which is a measure of the total time spent sleeping divided by the total time in bed [13], as well as periodic limb movement syndrome, characterized by repetitive movements of the limbs during sleep [14]. In their recently published meta-analysis that contained both objective and subjective studies, Wu et al. showed impaired sleep quality in patients with rheumatoid arthritis and recommended heightened attention to the care and management of sleep problems in these patients [10]. Similarly, a high prevalence of sleep disorders was reported for patients with ankylosing spondylitis (AS), with high co-occurrence of sleep disorders and AS-related problems, including depression, anxiety, and nocturnal pain [12]. Likewise, patients with psoriatic arthritis were found to have a higher incidence of sleep disturbances [15].

Obstructive sleep apnea (OSA) is an important sleep disorder that has been reported in individuals with rheumatological diseases, including SLE [16,17,18]. OSA is characterized by repeated episodes of upper airway collapse and obstruction during sleep associated with multiple sleep arousals and intermittent hypoxia. The obstructive event occurs due to the interaction of unfavorable anatomy of the upper airways and decreased muscle tone. The presence and severity of OSA are quantified by the Apnea Hypopnea Index (AHI), which reflects the number of obstructive respiratory events in an hour of sleep [19].

Previous studies that evaluated sleep disturbances in patients with SLE have mainly been based on subjective measures, such as sleep questionnaires, which can be prone to bias and inaccuracies. Few studies have used objective measures, such as polysomnography (PSG), which is considered the gold standard sleep examination. Multiple parameters are assessed during PSG, including an encephalogram, eye movements, an electrocardiogram, oxygen saturation, breathing rate and patterns, and limb movement [3,20,21,22].

Several studies employing PSG have found that patients with SLE have a higher occurrence of sleep disorders compared to healthy controls. Flores et al. assessed 14 patients with SLE and 11 healthy controls and found an increased presence of sleep disorders [22]. Iaboni et al. evaluated 35 patients with SLE and 17 healthy controls and reported that 20 out of 35 patients had OSA [3]. Increased prevalence of OSA was also described for rheumatoid arthritis patients [23]. In contrast, there is inconsistency in the current literature regarding the prevalence of OSA in ankylosing spondylitis. While some studies support the association between OSA and ankylosing spondylitis [18,24], others fail to establish a significant association between the two [25].

The association between OSA and a higher incidence of autoimmunity is complex. A possible explanation linking OSA and autoimmunity might be the presence of a chronic inflammatory state caused by repeated episodes of interrupted breathing and desaturation during sleep. Apneas causing chronic intermittent hypoxia may activate an inflammatory cascade leading to elevated cytokine levels, such as tumor necrosis factor alpha, interleukin 1β, interleukin 6, and interleukin 8, possibly contributing to autoimmunity. Alternatively, the chronic inflammatory state in an autoimmune disease might have a role in the excess risk of OSA [26,27].

In this study, we sought to investigate the relationship between OSA and SLE. We aimed to assess whether individuals with SLE have a higher prevalence of sleep disorders, specifically OSA, compared to a healthy control population. We also aimed to explore any potential predictors for OSA, such as disease activity, damage accumulation, and medications. Possible interactions with additional clinical features frequently observed in patients with SLE, such as fatigue, secondary fibromyalgia, and depression, were assessed as well.

## 2. Materials and Methods

### 2.1. Study Design

This is a single-center, cross-sectional, controlled study. The study group included consecutive patients with SLE at the Rheumatology Clinic of the Tel Aviv Sourasky Medical Center (TASMC), a tertiary-level hospital in Israel, between August 2020 and December 2020.

Consecutive patients who met the American College of Rheumatology (ACR) revised criteria for SLE and were 18 years of age or older were invited to participate in the study. Healthy controls were recruited from the Department of Rheumatology department employees and relatives. Healthy controls were matched to the study group based on sex, age, and body mass index (BMI). Women who were pregnant or had given birth in the year prior to enrollment were excluded, as were individuals with a known sleep disorder and those unable to provide informed consent. All of the patients and controls provided informed consent to participate, and the study was approved by the institutional review board (serial study number 59914).

### 2.2. Clinical Assessment

All study participants were asked to complete a series of validated questionnaires in order to assess various aspects of sleep and health-related quality of life. These questionnaires were selected based on their established reliability and validity in measuring the specific aspects of sleep and health that the study aimed to investigate.

The Epworth Sleepiness Scale (ESS) is a widely used questionnaire that assesses the general level of daytime sleepiness. The ESS questionnaire score ranges from 0 to 24, with higher scores indicating increased daytime sleepiness and a score above 10 considered to reflect excessive sleepiness [28].

The Pittsburgh Sleep Quality Index (PSQI) is a widely used self-rated questionnaire for assessing sleep quality and sleep disturbances. The PSQI questionnaire score ranges from 0 to 21, and a score above 5 indicates poor sleep quality [29].

The Functional Assessment of Chronic Illness Therapy (FACIT) measures fatigue on a scale of 0–52, with higher scores indicating increased levels of fatigue [30].

The Symptoms Severity Scale (SSS) and the Widespread Pain Index (WPI) questionnaire, which measures the number of painful body regions, are used to assess the presence of fibromyalgia. A combined SSS of ≥5 and a WPI of ≥7 is suggestive of fibromyalgia, and higher scores might be indicative of increased symptomatology of fibromyalgia [31].

The Beck Depression Inventory (BDI) is a widely used 21-item self-reporting questionnaire that evaluates the severity of depression in both normal and psychiatric populations, with higher scores indicating worse depression [32].

The assessment of SLE disease activity and damage accumulation in the current study group was performed with validated scoring systems, including the Systemic Lupus Erythematosus Disease Activity Index 2000 (SLEDAI-2K) and the Systemic Lupus International Collaborating Clinics/American College of Rheumatology (SLICC/ACR) damage index (SDI) [33,34].

Additional clinical data, including BMI, medication usage, and laboratory test results, were collected from the medical records of the study participants.

### 2.3. Assessment of Sleep Breathing Disorders

Each study participant was given the “WatchPAT 200”, a home sleep testing device, for a single night of testing. This device is a validated tool for the diagnosis of sleep disorders, including OSA, whose accuracy and reliability were demonstrated in previous studies [35]. The device is worn as a wristwatch and includes a finger probe and a sensor located on the patient’s chest. The finger probe measures oxygen saturation and the peripheral arterial tonometry (PAT) signal, which reflects vascular changes controlled by the sympathetic nervous system. A respiratory event is typically accompanied by transient peripheral vasoconstriction and an increased pulse. These physiological changes are recorded by the device and scored as apnea or hypopnea. An accelerometer is embedded in the wrist unit and monitors motion, while the chest probe detects snoring and body position [36]. The device is equipped with built-in software that automatically analyzes the data and generates a report that includes a variety of parameters related to sleep and respiratory function [37].

Some of the key parameters included in the report are:

- Oxygen saturation levels: measured by pulse oximetry;
- Respiratory events, including:

AHI—Apnea Hypopnea Index—a measure of the number of apnea events defined as a complete cessation of breathing for more than ten seconds, and hypopneas, which is a partial cessation of breathing leading to desaturation per hour of sleep [38];

RDI—Respiratory Disturbance Index—a wider definition than AHI that includes apneas, hypopneas, and respiratory effort-related arousal (RERA), which is a limitation in breathing that results in increased respiratory effort and culminates in arousal from sleep;

ODI—Oxygen Desaturation Index—defined as the number of episodes of oxygen desaturation in one hour of sleep, with oxygen desaturation considered a decrease in blood oxygen saturation to lower than 3% below baseline [39,40].

- Sleep/wake status: indication of whether the patient was awake or asleep during various periods of the night;
- The number of arousals: measurement of the number of times the patient had woken up or became partially awake during the night;
- Sleep efficiency: measurement of the percentage of time the patient spent asleep during the night;
- Actigraphy information: estimation of wakefulness and sleep according to the patient’s motion as measured by an accelerometer located on the patient’s wrist [41];
- Sleep stages: detection and determination of light sleep, deep sleep, and rapid eye movement (REM) sleep stages.

The severity of OSA is commonly measured by the AHI. An AHI of up to 4.9 per hour is considered normal. Mild OSA is defined as an AHI of 5 to 14.9, moderate OSA as an AHI of 15 to 29.9, and severe OSA as an AHI above 30 [42]. Sleep efficiency and the number of arousals per night were also used to assess the quality of sleep [13].

All participants in the study were informed of their results and were referred to a sleep clinic for further evaluation if needed.

### 2.4. Statistical Analysis

The statistical analysis for this study was conducted using R software version 4.0.2. The sample size was determined by considering a significance level of 0.05, a power of 0.8, and an expected effect of 0.4. The ratio between the study group and the comparison group was set to 2:1. Continuous data were reported using means and standard deviations, and they were compared between groups by the Mann–Whitney test. Categorical data were reported as frequency and percentage, and they were compared between the groups by the Fisher exact test. Both continuous and categorical data were also analyzed with the use of linear and logistic regression. All statistical tests were two-sided, and a *p*-value of less than 0.05 was considered statistically significant.

## 3. Results

### 3.1. Comparison Groups

The study cohort consisted of 42 patients with SLE and 20 healthy controls. The characteristics of the patients with SLE are described in Table 1. There were no significant differences in age, sex, or BMI between the study and the control groups (females 90.4% vs. 75%, *p* = 0.12; mean age 39.8 ± 10.8 vs. 38.1 ± 7.9 years, *p* = 0.49; and BMI 23.7 ± 4.4 vs. 22.8 ± 2.1, *p* = 0.51, respectively).

### 3.2. Quality of Sleep, Pain, and Fatigue

The study group reported worse sleep quality compared to the control group, as shown by higher scores on the PSQI questionnaire, increased fatigue as reflected by higher scores on the FACIT questionnaire, and more severe symptoms of fibromyalgia, as indicated by significantly higher scores on the WPI and SSS questionnaires. However, there were no group differences in the level of daytime sleepiness as measured by the ESS questionnaire (Table 2).

### 3.3. OSA Evaluation

**Figure 1:**
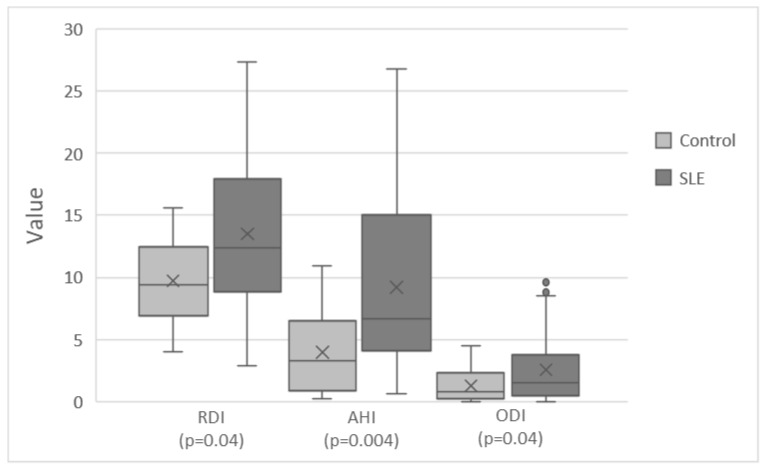
Sleep disturbance indexes in patients with SLE and healthy controls. The SLE group had increased AHI, ODI, and RDI scores compared to the control group (9.2 ± 6.8 vs. 3.9 ± 3.5, *p* = 0.004, 2.5 ± 2.7 vs. 1.3 ± 1.4, *p* = 0.04, and 13.5 ± 6.5 vs. 9.7 ± 3.6, *p* = 0.04, respectively). SLE, systemic lupus erythematosus; RDI, Respiratory Disturbance Index; AHI, Apnea Hypopnea Index; ODI, Oxygen Desaturation Index.

The SLE group had higher AHI, ODI, and RDI than the control group (Figure 1). It also had higher rates of sleep arousal and decreased sleep efficiency (7.7 ± 5.6 vs. 5.1 ± 2.3, *p* = 0.01 and 83.4 ± 6.1 vs. 87.2 ± 4.2, *p* = 0.03, respectively). Table 3 presents the results of the WatchPAT 200 sleep assessment.

The percentage of moderate-to-severe OSA (AHI ≥ 15) was significantly higher among patients with SLE compared with controls (23.6% vs. 0%, respectively, *p* = 0.045). Although the difference did not reach a level of significance, the patients with SLE had a greater number of periods of light sleep, less deep sleep, and less REM sleep compared to the control group. There were no group differences in the measured saturation indexes, including average saturation and minimal saturation levels.

A multivariate analysis with AHI as a dependent variable and significant or marginally significant univariate predictors as covariates (Table 4) revealed that only the SDI and BMI were associated with an elevated AHI (*p* = 0.03 and *p* = 0.01, respectively). The results of the multivariate analysis are shown in Table 5.

In a univariate logistic regression analysis, with moderate-severe OSA (AHI ≥ 15) as a dependent variable and all other variables as predictors, SLEDAI-2K was found to be positively associated with an AHI of ≥15 (*p* = 0.03).

No significant correlation was found between the AHI and the following parameters: sex, age, SLE disease duration, the presence of nephritis, the presence of anti-phospholipid antibodies, or specific medications, including corticosteroids. Interestingly, three patients had a history of neuropsychiatric SLE, and two of them had significant OSA with AHIs of 26.8 and 18.2. The subjective scores of the PSQI, ESS, FACIT, WPI, SSS, and Beck questionnaires were not found to correlate with an elevated AHI.

## 4. Discussion

This study aimed to objectively evaluate the presence of sleep-disordered breathing in patients with SLE by means of a sleep examination device. We also subjectively assessed the quality of sleep, daytime sleepiness, symptomatology of pain, fibromyalgia, fatigue, and depression. The results revealed an increased prevalence of OSA in patients with SLE compared to healthy controls. OSA severity, as assessed by measuring AHI, RDI, and ODI, was greater in patients with SLE, who had lower sleep efficiency scores and an increased number of arousals. Our evaluation of an association between OSA and other factors demonstrated that an elevated BMI, as well as SLE-related damage accumulation, as reflected by the SDI, were associated with a higher AHI. Increased SLEDAI-2K scores emerged as being associated with moderate-to-severe OSA.

Iaboni et al. conducted a study on a cohort of 35 patients with SLE, aiming to investigate the presence of sleep-related disorders. Those authors used PSG to evaluate the patients’ AHIs and found that 57% of the patients had an AHI > 5, which is indicative of sleep-related breathing disorders, such as OSA. We found a slightly higher OSA rate among patients with SLE, with 68% of patients having an AHI greater than 5. A more severe form of OSA, with an AHI equal to or greater than 10, was reported by Iaboni et al. in 26% of their cohort, similar to our finding of an AHI ≥ 15 in 23.6% of the patients with SLE [3]. Similarly, Flores et al. reported increased sleep-related breathing events in patients with SLE compared to healthy controls, although it was a smaller study of 14 patients [22]. In our study, the patients with SLE had more increased sleep arousal and poorer sleep efficiency compared to the healthy controls, a finding that may contribute to the increased fatigue reported by the SLE patients. Similar findings of greater sleep fragmentation in patients with SLE compared to healthy controls were reported by Moraleda et al. However, those authors assessed sleep fragmentation by actigraphy, which does not allow for the assessment of a possible contribution of OSA [21]. Unlike the findings of other studies, we did not observe a correlation between sleep-disordered breathing variables, fibromyalgia, or depressive symptomatology severity [6,17]. Further investigation of possible correlations between these elements in patients with SLE is warranted.

The findings of this current study demonstrate comparable rates of OSA in SLE patients to previously reported data on rheumatoid arthritis patients; in their PSG-based study, Wali et al. found an estimated OSA prevalence of 58.1%, with a prevalence of moderate-to-severe OSA of 22.9% [23]. In contrast, Solak et al. reported lower rates of OSA in patients with ankylosing spondylitis at 22.6% compared to our study [24].

We used the PSQI and the FACIT questionnaires to assess sleep quality and fatigue. The PSQI and FACIT questionnaire scores were worse in patients with SLE compared to the control group, reflecting the poorer quality of sleep and increased fatigue. There was no association with OSA contrary to our expectations. One possible reason for this could be the relatively small sample size of the study, which may preclude the detection of any significant associations. Moreover, PSQI and FACIT questionnaire scores reflect several factors, and they can be increased due to reasons other than OSA.

OSA has been well established as a risk factor for a wide range of cardiovascular diseases, including hypertension, coronary artery disease, arrhythmias, heart failure, and stroke. The association is thought to be due to the repetitive apnea events that expose the cardiovascular system to cycles of hypoxia, increase sympathetic activity, raise blood pressure, heart rate, and myocardial wall stress, provoke oxidative stress and systemic inflammation, cause endothelial dysfunction, and lead to other changes in the cardiovascular system [43]. Given the well-established association between OSA and cardiovascular risk, it is important to investigate the potential association between OSA and cardiovascular risk in SLE patients as well. Further research is needed to better understand this association.

Interestingly, our results have shown a significant association between SLE disease activity and damage accumulation with an increased risk of OSA. This finding is in line with previous reports in the literature [20,22]. However, the cross-sectional design of the present study as well as other published reports, does not allow conclusions regarding causality. Further research would be needed to establish causality. In the context of disease activity, it is noteworthy that our study cohort had a relatively low rate of glucocorticoid use (40%), reflecting the accepted practice at our center to aim for the lowest corticosteroid dose and cessation of steroids whenever feasible.

We believe that our findings of an association between OSA and SLE add important information to the current knowledge in several aspects. Increased awareness of the presence of OSA in patients with SLE is warranted in view of similar clinical manifestations shared by active SLE and OSA, such as fatigue, pain, and increased inflammatory markers. Thus, the detection of OSA may possibly prevent unnecessary treatment aimed at the management of a suspected flare of SLE. Moreover, the diagnosis and management of OSA may improve the quality of life of SLE patients. Increased awareness and early OSA detection are in accordance with previous studies assessing sleep disturbances in rheumatoid arthritis patients, as several studies have recommended considering OSA screening in patients with rheumatic diseases and referral to a sleep disorders clinic when needed [16,44].

Future studies addressing the optimal OSA management in these patients and its impact on SLE disease activity and fatigue are needed. Additionally, the increased cardiovascular risk present in both SLE and OSA emphasizes the importance of recognizing OSA in patients with SLE and possibly including OSA as an SLE comorbidity. Importantly, none of the sleep questionnaires predicted the presence of OSA; therefore, we suggest that physicians who treat SLE patients should consider performing an objective sleep assessment screening with a simple home device to allow early detection and enhanced management.

A limitation of our study was the use of the WatchPAT 200 device and not polysomnography, which is considered the gold standard for the assessment of sleep-disordered breathing and sleep architecture. However, since several studies have shown the validity of a diagnosis of OSA with the WatchPAT 200 device, we believe that its accuracy is appropriate for the purposes of this study [35]. In addition, movement sleep disorders, such as periodic limb movement syndrome, which was reported to be increased in SLE [3], could not be assessed with the WatchPAT 200 device. Despite these limitations, our ability to detect sleep-disordered breathing with a simple home device allows for a more accessible sleep evaluation.

Our study has several notable strengths that contribute to the current understanding of objective sleep disorder evaluation in patients with SLE. One major strength is the relatively large cohort size compared to those of previous studies, thereby allowing meaningful and comprehensive data analyses. This is particularly important given the limited body of research in this field. Another strength of our study is the use of a home sleep testing device, which may provide a simple and easily accessible screening test for OSA in patients with SLE.

## 5. Conclusions

The patients with SLE had an increased occurrence of OSA and poorer quality of sleep compared to healthy controls. Our findings suggest an association between OSA, SLE damage accumulation, and disease activity. The increased prevalence and severity of OSA in patients with SLE suggest that evaluation for this condition is important for patient management. In view of the poor correlation between subjective tools and objective findings of OSA in patients with SLE, we believe that objective screening of sleep assessment with a simple home device may allow early detection and management. Further larger studies are warranted to support our findings.

## Figures and Tables

**Table 1:** Characteristics of patients with SLE and healthy controls.

General Characteristics	SLE Group (*n* = 42)	Control Group (*n* = 20)	*p*-Value
Age (years)	39.8 ± 10.8	38.1 ± 7.9	0.49
Female, *n* (%)	38 (90.4)	15 (75)	0.12
BMI, Kg/m^2^	23.7 ± 4.4	22.8 ± 2.1	0.51
*Disease characteristics*			
Mean duration from SLE diagnosis (years ± SD)	12 ± 9.9	-	-
SDI (mean ± SD; median, range)	0.69 ± 1.09; 0, 0–5	-	-
SLEDAI-2K (mean ± SD; median, range)	3.45 ± 3.54; 2, 0–19	-	-
Patients with anti-phospholipid antibodies, *n* (%)	21 (50)	-	-
Patients with a history of lupus nephritis, *n* (%)	16 (38)	-	-
Patients with a history of neuropsychiatric lupus, *n* (%)	3 (7)	-	-
*Treatment modalities*			
Hydroxychloroquine, *n* (%)	39 (92.8)	-	-
Glucocorticoids, *n* (%)	17 (40)	-	-
Biologics ^¥^, *n* (%)	7 (16.6)	-	-
Immunosuppressives ^§^, *n* (%)	19 (45.2)	-	-

SLE, systemic lupus erythematosus; BMI, body mass index; SD, standard deviation; SDI, Systemic Lupus International Collaborating Clinics/American College of Rheumatology (SLICC/ACR) damage index; SLEDAI-2K, Systemic Lupus Erythematosus Disease Activity Index 2000. ^¥^ Biologic treatment modalities included belimumab and rituximab. ^§^ Immunosuppressive treatment modalities included mycophenolate mofetil, azathioprine, methotrexate, cyclophosphamide, and leflunomide.

**Table 2:** Analysis of the five questionnaires.

Questionnaires	SLE Group (*n* = 42)	Control Group (*n* = 20)	*p*-Value
PSQI	8.14 ± 3.47	5.10 ± 2.64	0.001
ESS	7.42 ± 5.50	5.78 ± 2.84	0.46
FACIT	16.89 ± 11.19	7.29 ± 5.93	0.0008
WPI	3.14 ± 4.48	0.55 ± 0.98	0.004
SSS	4.82 ± 2.96	1.38 ± 1.97	<0.0001

SLE, systemic lupus erythematosus; PSQI, Pittsburgh Sleep Quality Index; ESS, Epworth Sleepiness Scale; FACIT, Functional Assessment of Chronic Illness Therapy; WPI, Widespread Pain Index; SSS, Symptoms Severity Scale.

**Table 3:** WatchPAT 200 sleep parameters analysis.

Sleep Parameters	SLE Group (*n* = 42)	Control Group (*n* = 20)	*p*-Value
RDI	13.5 ± 6.5	9.7 ± 3.6	0.04
AHI	9.2 ± 6.8	3.9 ± 3.5	0.004
ODI	2.5 ± 2.7	1.3 ± 1.4	0.04
Sleep efficiency (%)	83.4 ± 6.1	87.2 ± 4.2	0.03
Number of arousals	7.7 ± 5.7	5.1 ± 2.3	0.01
Saturation (%)	96.1 ± 1.4	95.7 ± 1.4	0.45
Sleep latency	21.2 ± 9.3	18.5 ± 6.6	0.27
REM latency	87.9 ± 49.1	73.6 ± 46.2	0.09
REM	25.3 ± 14.0	27.3 ± 6.8	0.15
Light sleep	57.6 ± 12.4	52.7 ± 11.6	0.18
Deep sleep	17.9 ± 5.6	18.8 ± 6.7	0.54

SLE, systemic lupus erythematosus; RDI, Respiratory Disturbance Index; AHI, Apnea Hypopnea Index; ODI, Oxygen Desaturation Index; REM, rapid eye movement.

**Table 4:** Univariate analyses predicting AHI in patients with SLE.

Predictor	Estimate	Std. Error	t Value	*p*-Value
Age	0.178	0.103	1.729	0.09
Sex	1.235	3.635	0.34	0.74
BMI	0.521	0.234	2.229	0.03
SLE duration	0.116	0.109	1.059	0.30
Nephritis	2.586	2.245	1.152	0.26
aPL	3.378	2.166	1.56	0.13
Methotrexate	2.394	4.124	0.581	0.57
Azathioprine	1.825	3.291	0.555	0.58
Cyclophosphamide	4.011	6.948	0.577	0.57
Leflunomide	6.168	6.904	0.893	0.38
Biologic treatment ^¥^	1.88	2.865	0.656	0.52
Corticosteroids	3.511	2.21	1.589	0.12
Hydroxychloroquine	−4.175	4.955	−0.843	0.41
PSQI	0.223	0.326	0.684	0.40
ESS	−0.184	0.38	−0.773	0.45
FACIT	0.042	0.107	0.395	0.70
WPI	0.026	0.254	0.104	0.92
SSS	−0.033	0.386	−0.086	0.93
BDI	−0.19	0.16	−1.189	0.24
SDI	1.768	1.037	1.706	0.10
SLEDAI-2K	0.746	0.451	1.655	0.11

AHI, Apnea Hypopnea Index; BMI, body mass index; SLE, systemic lupus erythematosus; aPL, anti-phospholipid antibodies; PSQI, Pittsburgh Sleep Quality Index; ESS, Epworth Sleeping Scale; FACIT, The Functional Assessment of Chronic Illness Therapy—Fatigue; WPI, Widespread Pain Index; SSS, Symptom Severity Score; BDI, Becks Depression Inventory; SDI, Systemic Lupus International Collaborating Clinics/American College of Rheumatology (SLICC/ACR) damage index; SLEDAI-2K, Systemic Lupus Erythematosus Disease Activity Index 2000. ^¥^ Biologic treatment modalities included belimumab and rituximab.

**Table 5:** Linear multivariate model for the prediction of AHI in patients with SLE.

Predictor	Estimate	Std. Error	t Value	*p*-Value
(Intercept)	−6.599	5.554	−1.188	0.24
BMI	0.607	0.225	2.702	0.01
SDI	2.208	0.970	2.276	0.03

AHI, Apnea Hypopnea Index; SLE, systemic lupus erythematosus; BMI, body mass index; SDI, Systemic Lupus International Collaborating Clinics/American College of Rheumatology (SLICC/ACR) damage index.

## Data Availability

The data presented in this study are available on request from the corresponding author. The data are not publicly available due to privacy issues.
